# Chats about Medical Museums

**Published:** 1887-08-06

**Authors:** 


### CHATS ABOUT MEDICAL MUSEUMS.

By One of the Crowd.

(Continued from p. 191, vol. ii.)

VIII.—The London Hospital.

The museum in connection with the London Hospital is to be found in the fine new building opened last October for the medical college attached to that institution. The chamber in which the anatomical and histological collections are displayed has been built specially for the purpose, and is well adapted to it, and some day the museum altogether will no doubt be among the most interesting in London. At present it can hardly be said to be complete. For a great portion of it there is no catalogue at all, and the descriptive labels attached to many objects have faded into complete illegibility, and the beholder has to evolve his notions of things very much as the famous German metaphysician did his camel.

There is a large ground-floor, and a single gallery running all round the museum, the whole being well lighted from above. In the glass cases lying round the front of the gallery bare bones are arranged with a systematic order, and are labelled with a minuteness which, if they do not render osteology as diverting and enjoyable as beer and skittles, nevertheless seem to simplify the science considerably. All the rest of the objects in the gallery are bottled specimens of one thing and another, and to the benighted ignoramus who comes to their inspection with no special knowledge of physiological matters, they are not very instructive or exhilarating. The gentleman who said he was a happy man till he found out that he had got a liver, gave expression to that which may be a useful warning. "Wish to goodness I'd never seen the thing!" said an unhappy patient to his family doctor the other day as he glowered savagely at a handsomely bound volume of "The Family Æsculapius," or something of the kind, which he had been taking in in weekly instalments. From the very first part he and his family had never been quite free from some alarming symptom or other. Similarly, one may settle down into a condition of the dolefulest of dumps by scrutinising a long series of degenerate hearts and hardened livers, phthisical lungs and diseased kidneys. The poet who talked about our being "harps of a thousand strings," and wondered that we should keep in tune so long, could never have been through a good anatomical museum or he would have made it ten thousand at the very least, even though his verse should have limped a little. A thousand strings indeed! Why, every sinner of us is a Handel Festival orchestra, and the wonder is that we are ever in tune at all. Something like that is the reflection suggested at every turn the unfamiliar observer takes in a place of this kind, and he is apt to leave such a collection of "preparations" as are here displayed with a feeling not altogether unlike that of the madman who believed himself to be made of glass, and who was continually haunted by the fear that he would break himself.

Some of the tissues displayed here by means of injections of carmine, or vermilion, or mercury, are exquisitely beautiful. This injection is performed to render distinguishable the tiny tubes with which every atom of the body is pervaded. There is one bottle here containing a portion of the small intestines in which the lacteals have been injected with mercury, the arteries with some red fluid, and the veins with brown. The mercury shows yellow, and every minute tube through which it has been forced gleams out as a fibre of dead gold. There are several objects treated in this way with an exquisitely beautiful effect.

"The heart will break, yet brokenly live on," says Byron. It may be true in the poetic sense of the words a broken heart, but in the doctor's sense it is certainly not true. There are several broken hearts here—literally broken, and presenting great rents in them, just such as might be expected when disease has rendered the heart too thin and weak to resist the pressure exerted in driving the blood through the entire system of the body. Is there, it occurs to one to ask, any basis of physical fact for the idea expressed in the common allusion to the breaking of the heart? When a person dies of a broken heart, as poets and novelists have taught us to believe they sometimes do, is the heart ever literally fractured? Has either of these ruptured organs ever belonged to a deserted lover, or a ruined adventurer, or a bereaved parent? Possibly it may. By the broken heart ordinarily is meant, or should be, a broken spirit—a spirit out of which all hope and energy have been crushed by overwhelming trouble or calamity. But trouble or calamity may sometimes come with a fearful inrush of emotion, which sets up what the doctors call violent cardiac action, that the structure of the heart may not be able to stand. Most medical museums have examples of rupture of the heart brought about in this way, and in such cases, of course, it is literally correct to say that a broken heart was the cause of death.

Among minor items of this collection on the ground-floor are some odd things in the shape of fœtal snakes and cuttle-fish, a dissection showing the nervous and digestive system of the common earth-worm, the intestines of a cat, the brain of an alligator, and a number of other things trivial enough in themselves, but all of them, it may be presumed, valuable as illustrations of some phase or other of the great and infinitely complex science of animal physiology. Over the shelves bearing these minor objects there have been laid up out of the way, portions of skeletons, which seem to be grinning down on the visitor even more hideously than their fellows formally adjusted in glass cases, especially as, lying up there in a new building and near a new wall, they have grown somewhat mouldy. This is the case, indeed, with a good many of the objects in the collection. There are two or three preserved heads of savages, one of a Chinese and another of a tattooed Maori, to which a slight covering of blue mould imparts an aspect ghastly beyond expression.

The models in this museum are not specially admirable, though they are the most striking objects in the place. There are, however, a series of dissections of portions of the human body preserved in spirit, which are quite evidently masterpieces of their kind, and which must be infinitely more instructive than such models as stand near them, and to which, indeed, the best artificial productions can hardly be compared. Near one of the doors is a picture of an intelligent-looking Oriental, shown full front and in profile, and which in some other places would suggest merely a freak of the artist's fancy. Seen here, however, it must be presumed to have some claim to be regarded as a faithful representation of a genuine *lusus naturæ*. The man has a supplementary pair of legs growing out of his stomach, and in one of the glass cases is a small coloured figure of a Chinaman with some similar addition to his anatomy.

There are yet one or two other museums to which a future notice must be given.

*(To be continued.)*

